# HSV-1 miR-H6 Inhibits HSV-1 Replication and IL-6 Expression in Human Corneal Epithelial Cells In Vitro

**DOI:** 10.1155/2012/192791

**Published:** 2012-04-09

**Authors:** Fang Duan, Jingyu Liao, Qiang Huang, Yuhong Nie, Kaili Wu

**Affiliations:** ^1^Zhongshan Ophthalmic Center, State Key Laboratory of Ophthalmology, Sun Yat-sen University, 54 Xianlie Road, Guangzhou 510060, China; ^2^Department of Ophthalmology, Renmin Hospital, Wuhan University, Wuhan 430060, China

## Abstract

HSV-1 infection in the cornea could lead to blindness. The infected cell polypeptide 4 (ICP4) of herpes simplex virus 1 (HSV-1) is a regulator of viral transcription that is required for productive infection. It has been previously demonstrated that miR-H6 encoded from HSV-1 genome targets ICP4 to help maintain latency. In this study, synthesized miR-H6 mimics were transfected into HSV-1-infected human cornea epithelial (HCE) cells. The inhibition of HSV-1 replication and viral ICP4 expression in miR-H6-transfected HCE was confirmed by plaque assay, immunofluorescence, and Western blot. Compared to nontransfection or mock, miR-H6 produced a low-titer HSV-1 and weak ICP4 expression. In addition, miR-H6 can decrease the interleukin 6 released into the medium, which was determined by ELISA. Taken together, the data suggests that miR-H6 targeting of ICP4 inhibits HSV-1 productive infection and decreases interleukin 6 production in HCE, and this may provide an approach to prevent HSV-1 lytic infection and inhibit corneal inflammation.

## 1. Introduction

Herpes simplex virus type 1 (HSV-1) is a linear double-stranded DNA virus that mainly infects epithelial and neuronal cells. The productive infection of HSV-1 entails a cascade of gene expression (immediate-early, early, and late genes), viral DNA replication, assembly and egress of virus. The immediate-early (IE) gene ICP4 (infected-cell polypeptide 4) of HSV-1 is one of the major regulatory genes required for efficient transcription of early and late viral genes and drives HSV-1 through the productive replication cycle [1, 2]. ICP4 also plays a role in reactivation of HSV-1 from latency. The regulation of ICP4 seems to be exerted at the posttranscriptional level by the latency-associated transcripts (LATs) [3, 4]. Inhibition of ICP4 gene can suppress viral production in various cells [5–8].

MicroRNAs (miRNAs) are small, 21~23 nucleotide non-coding RNAs that play an important role in the post-transcriptional regulation of gene expression in a wide range of organisms from unicellular eukaryotes to multicellular eukaryotes by a variety of mechanisms [9]. The existence of viral miRNAs was first reported in Epstein-Barr virus in 2004 [10]. The discovery of miRNAs encoded by viruses suggests that viruses have evolved to exploit RNA silencing for regulation of their own genes, host genes, or both and contribute to the functions including (I) latent and lytic viral infection, (II) immune evasion, (III) prevention of apoptosis, (IV) viral replication, and (V) others [11–13]. As for HSV-1, the first microRNA, miR-H1, expressed as a late gene in productive infection, was reported in 2006 [14]. Also, HSV-1 infection of human brain cells inducing a miRNA-146a that mediates inflammatory signaling was firstly reported in 2009 [15]. Up to now, 16 HSV-1 miRNAs have been found [4, 12, 14, 16]. Of them, both miR-H1 and miR-H6 are located upstream to the LAT promoter. miR-H1 is encoded by sequences upstream of the LAT promoter in the LAT sense direction, while miR-H6 is located in an LAT antisense direction [16]. Umbach et al. first found that miR-H6 downregulates the expression of ICP4 proteins, suggesting a contribution to the establishment and maintenance of viral latency [4]. Similarly, the latent infection is characterized by the abundantly expressed locus that encodes the LAT, which can repress lytic replication and IE gene expression in a neuronal cell line [3]. However, miR-H6 and H1, detected as early as 2 hr postinfection (p.i.) in cultured cells, were abundantly expressed in the HSV-1 productive infection sample, whereas the other HSV-1 miRNAs (i.e., miR-H2, -H3, and -H4) were greatly produced during the latent infection [16, 17]. Meanwhile, miR-H6 expression was significantly reduced in cells infected with a mutant HSV-2 virus with an insertion of a sequence between the LAT promoter and miR-H6 [18], and also in latently, but not in acutely, infected mouse ganglia with HSV-1 LAT deletion mutants (dlLAT1.8) [17]. These results suggested that when miR-H6 is expressed abundantly during lytic infection, it may play other roles during viral infection besides establishing and maintaining the latent infection [12, 17].

As a major pathogen to human beings, HSV-1 can cause a variety of diseases and can even be life threatening [19, 20]. Common ocular manifestations of HSV-1 infection include blepharitis, conjunctivitis, keratitis, and uveitis; of them, recurrent viral keratitis can lead to severe corneal blindness [2]. In addition, the cornea tissue was suggested to be the site where HSV-1 can establish latent infection [2, 21, 22]. The corneal epithelium is composed of several layers of cells in front of the stroma. Thus, it is given the important role of protecting from the invasion of exogenous viruses. The primary infection of HSV-1 in corneal epithelial cells is typically displayed by dendritic lesions. However, HSV-1induced keratitis also frequently appeared, as the immunopathology of inflammation was not directly produced by a great number of lytic infection of virus [23–26]. In such cases, cytokines play important roles in the virus-induced immunopathology. One cytokine known to contribute to immune response to HSV-1 is interleukin-6 (IL-6), which is a cytokine with pleiotropic activities, including both proinflammatory and anti-inflammatory activities [27]. Meanwhile, within the LAT and ICP0 promoter of HSV-1, there are IL-6 response elements, possible binding sites of the IL-6-induced transcription factors. Viral constructs with deletion of the IL-6 response element in the LAT promoter reactivate at much lower rates than similar constructs without the deletion [28]. IL-6 induced by HSV-1 infection has been reported in various cells such as leukocytes [29], epithelial cell EMT-6, and HaCat [30, 31], as well as cornea epithelial cells and fibroblasts [32].

Regarding the abundant expression in lytic infection and the location of miR-H6 encoding sequence (upstream of LAT promoter), we wonder whether miR-H6 has different functions compared to other miRNAs (miR-H2-miR-H5) in HSV-1 infection. We therefore investigated the effects of miR-H6 targeting of ICP4 on HSV-1 replication, in particular, the effects on IL-6 production in human cornea cells. We found that miR-H6 targeting of ICP4 suppresses HSV-1 replication and decreases IL-6 production in HCE, and this may provide an approach to prevent HSV-1 lytic infection and inhibit inflammation in cornea.

## 2. Materials and Methods

### 2.1. Cells and Virus Infection

HCE used in this study was derived from human limbal cells as described previously [33, 34]. Cells were cultured in DMEM/high-glucose supplemented with 10% fetal bovine serum (FBS; Gibco, NY, USA), 10 ng/mL human epidermal growth factor (EGF; Sigma, St. Louis, MO), 5 *μ*g/mL human transferrin (Sigma), 5 *μ*g/mL insulin, and 0.4 *μ*g/mL hydrocortisone (Gibco BRL, Grand Island, NY). HEp-2 cells were grown in DMEM/F12 with 10% newborn bovine serum (Gibco, NY, USA). The cells were incubated at 37°C in a 5% CO_2_-95% air incubator. Stocks of the HSV-1 (F strain) were propagated on HEp-2 cells, and the titer of virus stocks was determined according to a previously described method [35].

HCE cells were infected by HSV-1 using a previously described method [5, 34]. Briefly, cells were cultured to 80 to 90% confluence, then infected with HSV-1 for 1 h, with gentle 15 sec shaking every 15 min to allow viral absorption. After 1 h, the inoculum was removed and the medium was replaced with serum-free DMED/high-glucose. Cells infected at a multiplicity of infection (MOI) of 5, 1, 0.1, were washed with phosphate-buffered saline (PBS) three times and harvested.

### 2.2. HSV-1 miR-H6 Mimics and Transfection

The miR-H6 mimics we used were double-stranded, chemically synthesized by Guangzhou RiboBio Co., Ltd. (Guangzhou, China) according to the mature miR-H6 (5′-CACUUCCCGUCCUUCCAUCCC-3′). The mock is a small RNA that does not target any known gene as a negative control. The HCE cells were grown to 70–80% confluence and then transfected with miR-H6 (50 or 100 nM) and mock, by Lipofectamine 2000 (Invitrogen, Carlsbad, CA, USA), according to the manufacturer's instructions. Then, the cells are infected with HSV-1 at MOI 0.1 24 h posttransfection. At the indicated time, the cells and medium were harvested for further experiments.

### 2.3. Plaque Assays

The plaque assay was performed as we described previously, but with modification [5]. HCE cells were grown in 12-well plates to 70 to 80% confluence and then transfected with miR-H6 mimics and infected with HSV-1 as described above. After absorbing the HSV-1 for 1 h, cells were overlaid with 1 mL of a 1 : 1 mixture of low-melting-temperature agarose (NuSieve GTG Agarose, USA) and 2 × DMEM/high glucose to permit only cell-to-cell spread of virus. At 48 h p.i., agarose was removed carefully and plates were stained with crystal violet for 20 min and then photographed. Finally, plaque sizes were measured.

### 2.4. Real-Time PCR Analysis

HCE cells were cultured to 80 to 90% confluence and then infected with HSV-1 in a multiplicity of infection (MOI) of 5, 1, and 0.1. At 24 h p.i., cells were washed with PBS three times and harvested. Cellular total RNA was isolated with TRIzol reagent (Invitrogen, Carlsbad, CA, USA) according to the manufacturer's instructions. Subsequently, RNA samples were detected using an All-in-One miRNA Q-PCR Detection kit (FulenGen, Guangzhou, China). The miR-H6 5′ primers were CACTTCCCGTCCTTCCATCCCA (product size is 74) and homo snRNA U6 5′ primers were CAAATTCGTGAAGCGTTCCATAT (product size is 79). The reaction conditions were 95°C for 10 min, 38 cycles of 95°C for 10 s, 65°C for 20 s, and 72°C for 10 s and 72°C for 5 min by iQ5 Real-Time PCR Detection System (Bio-Rad). The amount of each mRNA was calculated relative to the amount of U6 mRNA in the same samples by iQ software. Each run was completed with a melting curve analysis to confirm the specificity of the amplification.

### 2.5. Indirect Immunofluorescence

At 12 and 24 h p.i., slide-mounted cells were used for indirect immunofluorescence analysis according to the method described previously [5]. Cells were incubated with mouse anti-human monoclonal antibody that recognizes HSV-1 ICP4 (Abcam, Cambridge, UK) at 4°C overnight. Cells were then incubated with TRITC-conjugated secondary goat anti-mouse IgG antibody (Zhongshan Goldenbridge, Beijing, China) at 37°C for 1 h. The nuclei were stained with hoechst for 10 min. Cells were then observed using a confocal laser scanning microscope (Carl Zeiss, Jena, Germany). Cells incubated with PBS (instead of the first antibody) were used as negative controls.

### 2.6. Western Blot Analysis

 At 24 h p.i., cells were lysed with lysate buffer (20 nM tris-HCL). The samples were freeze-thawed 3 times and then centrifuged at 12,000 rpm for 30 min at 4°C to remove cellular debris. Protein content in the supernatant was determined by the bicinchoninic acid method using BSA as the standard. Western blot was conducted according to our previous method [5]. The membranes were incubated with 1 *μ*g/mL of mouse anti-human monoclonal antibodies that recognizes HSV-1 ICP4 (Abcam, Cambridge, UK) or 0.2 *μ*g/mL mouse anti-GAPDH (KangChen, Shanghai, China) separately at 4°C overnight. Then, they were exposed to a secondary goat anti-mouse IgG antibody (Zhongshan Goldenbridge, Beijing, China) for 1 h. Protein bands were visualized with a kit of chemiluminescence Phototope (R)-HRP Western Blot Detection System (Cell Signaling Technology, Inc., Danvers, MA, USA) and exposed by Kodak Imaging Station 4000MM (Kodak, Rochester, NY, USA).

### 2.7. Enzyme-Liked Immunosorbent Assay (ELISA)

The HCE cells were transfected with miR-H6 and mock and then infected with HSV-1 as described above, or directly cultured the transfected cells without virus infection. The medium was collected at 6, 12, and 24 h p.i., or 30, 36, and 48 h post transfection for cells without HSV-1 infection, and the IL-6 levels in supernatant were determined using a specific IL-6 ELISA kit (Boster, Wuhan, China). Human IL-6 was used to construct a standard curve according to the manufacturer's instructions.

### 2.8. Statistical Analysis

All experiments were repetitively conducted a minimum of three times, and the quantitative data was expressed as means ± SD. Statistical analysis of data was performed by one-way ANOVA (SPSS 17.0), with *P* < 0.05 considered statistically significant.

## 3. Results

### 3.1. miR-H6 Effects on Cells and Viral Replication

**Figure 1:**
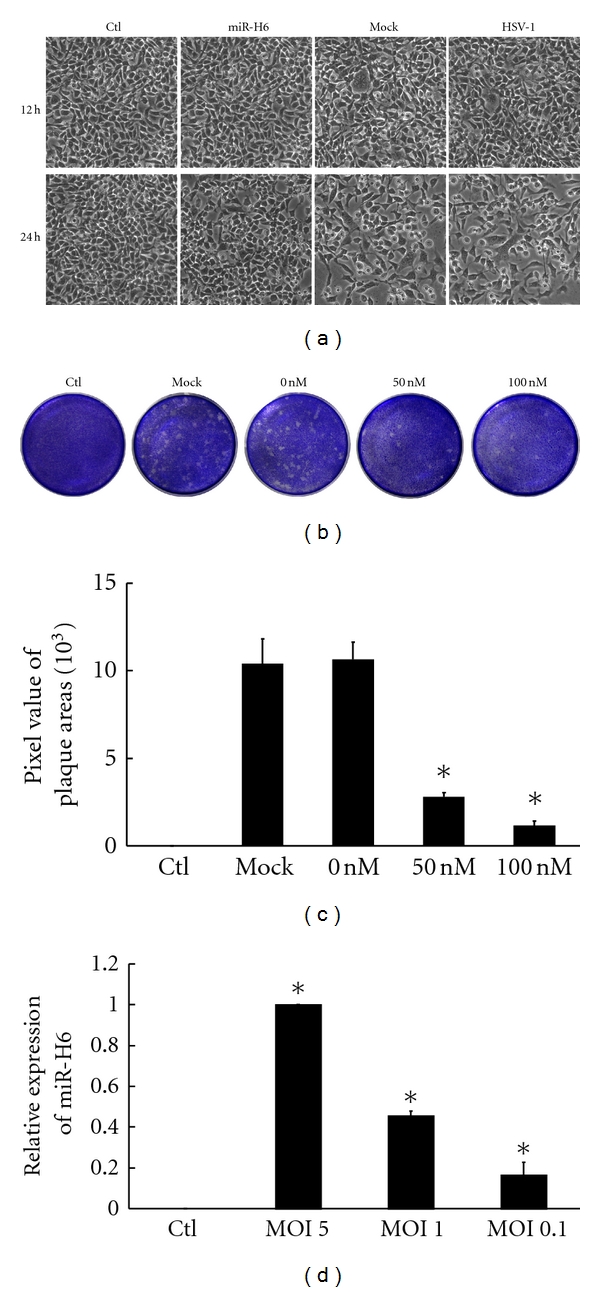
miR-H6 effects on cell changes and viral replication in HSV-1-infected HCE cells. HCE cells were transfected miR-H6 mimics and mock, followed by infection with MOI 0.1 of HSV-1 24 h posttransfection. (a) The cellular morphological changes were imaged at 12 h and 24 h postinfection. CPE clearly increased, and many multinucleated giant cells could be seen in the cells treated with mock RNA and cells untreated with small RNA; fewer CPE and giant multinucleated cells could be seen in cells transfected with miR-H6 mimics. (b, c) Plaque assay showed that treatment with miR-H6 decreased the plaque sizes and numbers and had a statistically significant difference (*P* < 0.05). Cells were treated with miR-H6 mimics (0, 50, 100 nM) and mock RNA (mock) or without small RNA (HSV-1). The control group (Ctl) consisted of cells without virus infection and small RNA treatment. (d) Real-time PCR determined miR-H6 expression in pooled HSV-1-infected HCE cells, and miR-H6 levels were changed in a viral-titer-dependent manner (*P < 0.01, one-way ANOVA, among MOI 0.1, 1, and 5).

HCE cells were transfected and infected HSV-1 at MOI 0.1 as described above, and cellular morphological changes were observed at 6, 12, and 24 h p.i. under phase-contrast microscopy (Figure 1(a)). At 6 h p.i., there were no obvious differences in cellular morphology among cells transfected with miR-H6, the mock, without small RNA, and control cells (data not shown). At 12 h p.i., the cytopathic effect (CPE) could be observed in the mock and without small RNA cells, but not in the cells transfected with miR-H6 and control. Infected cells usually displayed clusters, and many individual cells remained uninfected. At 24 h p.i., CPE increased dramatically, and many giant, multinucleated cells could be seen in the mock and without small RNA cells. However, fewer CPE could be seen in cells transfected with miR-H6, including multinucleated giant cells.

Plaque assay was used to detect the virus quantity in MOI 0.1 HSV-1-infected HCE cells and demonstrated that normal cell had no formation of plaque, and HSV-1 without small RNA resulted in the formation of a larger number of plaques, which was similar to the mock. However, treatment with miR-H6 significantly decreased the plaque sizes and numbers (Figure 1(b)) and had significant difference (Figure 1(c), *P* < 0.05). Meanwhile, miR-H6 was measured in HSV-1-infected HCE with MOI 5, 1, and 0.1, at 24 h p.i. The result showed that miR-H6 increased significantly in HSV-1-infected HCEs, and the expression of miR-H6 was consistent with the concentration of the infectious virus quantity (Figure 1(d), *P* < 0.01). The uninfected HCE cells had no expression of miR-H6.

### 3.2. miR-H6 Suppresses ICP4 Protein Expression

**Figure 2:**
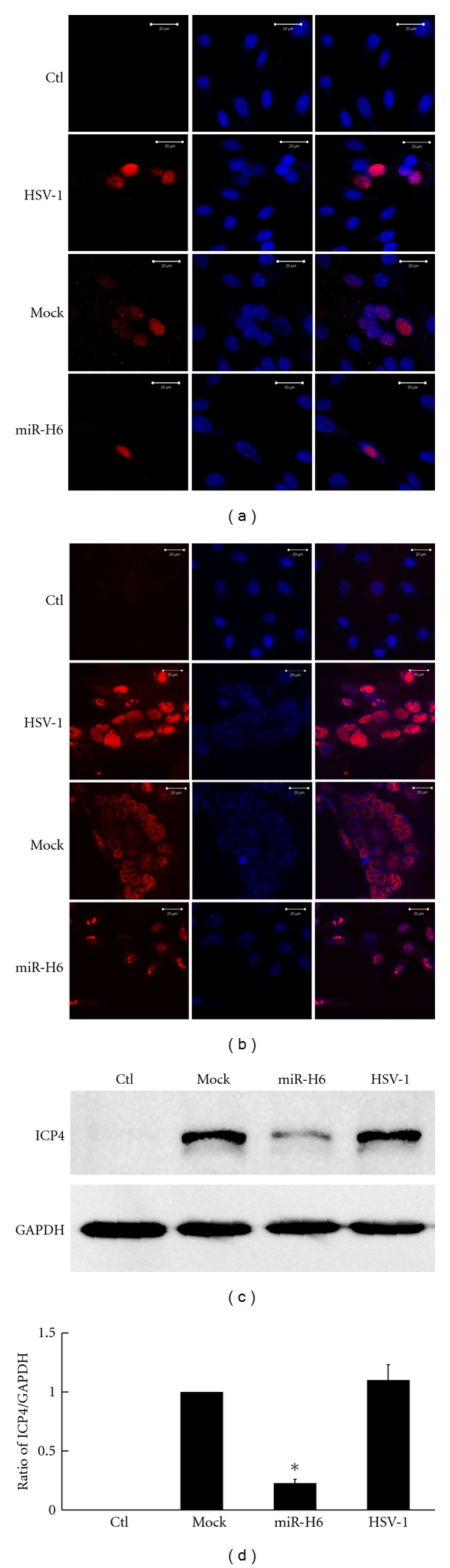
miR-H6 decreased ICP4 protein expression in HSV-1-infected HCE cells. HCE cells were transfected with miR-H6 mimics and mock RNA and then infected with MOI 0.1 of HSV-1 24 h posttransfection. The staining of ICP4 protein in HCE cells at 12 h p.i. (a) and at 24 h p.i. (b) TRITC labeled the antibody-stained ICP4 (red, left) and the hoechst-dyed nucleus (blue, middle). Images of ICP4 and the nucleus were merged (right), scalebar: 20 *μ*m. (c) The ICP4 band was determined by Western blot. (d) Quantitative analysis of bands in (c). Significantly lower levels of ICP4 in HCE cells transfected with miR-H6 (*P < 0.05, miR-H6 versus mock or HSV-1 alone).

First, the ICP4 protein expression was observed by indirect immunofluorescence at 12 and 24 h p.i. Following HSV-1 infection and up to 12 h p.i., there was very weak ICP4 staining in HCE cells transfected with mock, and without mall RNA, less ICP4 expression was noted in HCE cells transfected with miR-H6; ICP4 was not observed in normal HCE control cells (Figure 2(a)). At 24 h p.i., the ICP4 protein expression increased dramatically. Compared with the cells transfected with mock and cells without small RNA, the intensity of immunostaining for ICP4 was dramatically weaker in the HCE cells transfected with miR-H6 (Figure 2(b)).

The effect of miR-H6 on the ICP4 protein was also detected by Western blot using antibodies against ICP4 (Figure 2(c)). At 24 h p.i., the expression of ICP4 decreased in the lysate of HCE cells transfected with miR-H6, whereas the expression in small RNA untreated cells and in mock remained constant (Figure 2(d), *P* < 0.05). Similarly, there was no expression of ICP4 in normal HCE cells.

### 3.3. The Effect of miR-H6 on IL-6 Release

**Figure 3:**
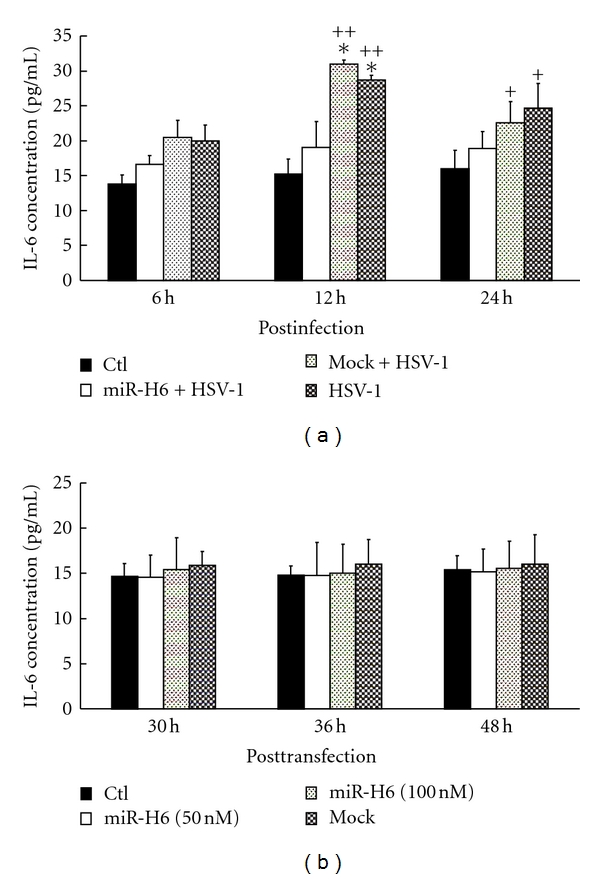
The effect of miR-H6 on IL-6 release in medium. HCE cells were transfected with miR-H6 and infected with MOI 0.1 of HSV-1 after 24 h posttransfection (a) or without viral infection (b). The IL-6 level in medium was measured at indicated time p.i. by ELISA. *Statistical difference between HCE cells transfected with miR-H6 mimics and mock or HSV-1 alone (*P* < 0.01); ^+/++^statistical difference between normal HCE cells and mock or HSV-1 alone (^+^ *P* < 0.05; ^++^ *P* < 0.01).

The IL-6 protein expression in the medium was determined by ELISA (Figure 3). At 6 h p.i., the IL-6 level in medium of HCE cells transfected with miR-H6 was lower than that with mock and without small RNA; however, there was no significant difference (*P* > 0.05). At 12 h p.i., the IL-6 level in HCE cells transfected with miR-H6 was dramatically lower than in those transfected with mock or without small RNA (Figure 3(a), *P* < 0.01). At 24 h p.i., the IL-6 levels in HCE transfected with mock and without small RNA decreased as compared to those at 12 h p.i. The IL-6 level in medium of cells transfected with miR-H6 is insignificantly lower when compared to the level of mock transfection or that of only HSV-1-infected cells. The latter two showed significantly higher levels as compared to the control (*P* < 0.05).

The effects of miR-H6 on IL-6 production of HCE cells were assayed by comparison of the IL-6 levels in HCE cells transfected with miR-H6 and mock and normal cells (Figure 3(b), *P* > 0.05). IL-6 did not alter significantly among all the groups at various time points.

## 4. Discussion

During HSV-1 lytic infection, ICP4 upregulates the early and late genes, downregulates the IE genes through interaction with the transcription factors associated with RNA polymerase II, and represses the LAT promoter and prevents LAT production [36, 37]. Also, ICP4 is the target of HSV-1 miR-H6 [4]. Therefore, we began this study with the goal of observing the effect of miR-H6 on HSV-1 replication and the inflammatory cytokine IL-6 production in HCE, by which HSV-1 infections can lead to cornea blindness [21, 38]. We found that miR-H6 suppressed the expression of ICP4 protein and inhibited the HSV-1 productive infection in HCE and in human retinal pigment epithelial cells (data not shown). A similar result was reported by Umbach et al., who found that miR-H6 inhibits expression of ICP4 in 293T cells cotransfected with a synthetic miR-H6 duplex intermediate and with plasmids expressing either wild-type ICP4 or the ICP4 mutant [4]. Because viral miRNA regulation is dependent on the context of infection, such as cell-type and viral genome expression, we focused on the HSV-1-infected HCE to investigate miR-H6 roles on the productive infection.

The highly abundant expressions of microRNAs in HSV-1 latent infection were found in previous studies, which led to the concept that microRNA plays important roles in establishing and/or maintaining latent infections [4, 16, 39]. However, by experiments in acute and latent ganglionic infection in mice and lytic infection in Vero cells infected with wide-type or LAT deletion mutant HSV-1, Kramer et al. found that LAT deletion mutants establish and maintain latent infections and concluded that microRNAs are not essential for latency in mouse trigeminal ganglia, but may help promote it [17]. Du and colleagues found that the expression of viral genes in explanted ganglia was disordered rather than sequentially ordered as in infected cells in culture, and the accumulation of viral mRNAs takes place concurrently with a decrease in the accumulation of miRNAs/LATs, which was degraded by a viral gene product other than the short half-life of these miRNAs/LATs [40]. Nevertheless, studies on quantities of HSV-1 mircroRNAs revealed that miR-H6 and miR-H1 in the productive infection were the most abundant, whereas miR-H2, -H3, and -H4 were greatly expressed in the latent samples [4, 16, 17]. We also revealed that the expression of miR-H6 in HSV-1-infected HCE cells increases with viral concentration. All these data suggest that HSV-1 microRNAs may have stage-specific functions in the virus life cycle and that highly abundant levels of miR-H6 may have other roles in lytic infection other than maintaining the latent infection [17]. In our present study, we are interested in miR-H6 silencing ICP4 by transfecting synthesized miR-H6 mimics into HCE cells and followed by HSV-1 infection. More studies on miR-H6, such as interference with its function, and precise analysis of time-dependent effects in lytic infection would have a greater effect on our understanding of the virus and its induced diseases.

HSV-1 infection in cornea is characterized by recurrent viral lytic infection, immune inflammation, neovascularisation, and so on. IL-6, as an important cytokine, targets multiple cell types and induces a broad array of responses. These responses are often, simplistically, classified as pro-or anti-inflammatory in nature [41]. It was showed that two major signal transduction pathways regulated by IL-6: STAT3 and SHP2/Gab/MAPK signaling, which involve the gp130 YxxQ and Y759 motives, respectively [42]. IL-6 plays important roles in these corneal pathological changes, which has been evidenced by previous studies [38, 43–46]. Also, HSV-1 infection influencing IL-6 has been previously reported. By using HSV-1 mutants lacking the virion-transactivating protein VP16 or any of ICP0, ICP4, ICP8, or ICP27, virus-infected leukocytes or murine epithelial cell line EMT-6 displayed unaltered capacities to induce IL-6 [29, 30]. Thus any one of them alone is not essential for IL-6 production. It was reported recently that in Kaposi's sarcoma-associated-herpes-virus-(KSHV-) infected cells, miR-1293, targeting viral IL-6 RNA, and miR-608, targeting human IL-6 ORF, can mechanistically compete with KSHV ORF57 protein, which binds RNA export factor (REF) at the same site and in a similar way to ICP27, and thus influence IL-6 production [47, 48]. Although in the present study we did not know if there was a target sequence of miR-H6 in HCE, we did not find miR-H6 suppressing IL-6 release in HCE without HSV-1 infection. Therefore, the miR-H6 decreasing IL-6 production in HSV-1-infected HCE cells is due to the suppression of virus production through inhibiting ICP4, because a functional viral genome was required for induction of IL-6 [29].

Our findings are that miR-H6 mimics could inhibit HSV-1 productive infection in HCE cells as well as accompanied IL-6 release. Silencing viral microRNA targets as a novel antiviral therapy, our result may imply that in diseased tissue like keratitis, using miR-H6 could not only suppress viral replication, but also decrease inflammation due to IL-6 production.

##  Conflict of Interests

There is no commercial conflict of interests or any other conflicts of interests in this paper.
